# Neural Mechanisms Underlying the Computation of Hierarchical Tree Structures in Mathematics

**DOI:** 10.1371/journal.pone.0111439

**Published:** 2014-11-07

**Authors:** Tomoya Nakai, Kuniyoshi L. Sakai

**Affiliations:** 1 Department of Basic Science, Graduate School of Arts and Sciences, The University of Tokyo, Komaba, Meguro-ku, Tokyo, Japan; 2 Japan Society for the Promotion of Science, Kojimachi, Chiyoda-ku, Tokyo, Japan; 3 CREST, Japan Science and Technology Agency, Goban-cho, Chiyoda-ku, Tokyo, Japan; University College London, United Kingdom

## Abstract

Whether mathematical and linguistic processes share the same neural mechanisms has been a matter of controversy. By examining various sentence structures, we recently demonstrated that activations in the left inferior frontal gyrus (L. IFG) and left supramarginal gyrus (L. SMG) were modulated by the Degree of Merger (DoM), a measure for the complexity of tree structures. In the present study, we hypothesize that the DoM is also critical in mathematical calculations, and clarify whether the DoM in the hierarchical tree structures modulates activations in these regions. We tested an arithmetic task that involved linear and quadratic sequences with recursive computation. Using functional magnetic resonance imaging, we found significant activation in the L. IFG, L. SMG, bilateral intraparietal sulcus (IPS), and precuneus selectively among the tested conditions. We also confirmed that activations in the L. IFG and L. SMG were free from memory-related factors, and that activations in the bilateral IPS and precuneus were independent from other possible factors. Moreover, by fitting parametric models of eight factors, we found that the model of DoM in the hierarchical tree structures was the best to explain the modulation of activations in these five regions. Using dynamic causal modeling, we showed that the model with a modulatory effect for the connection from the L. IPS to the L. IFG, and with driving inputs into the L. IFG, was highly probable. The intrinsic, i.e., task-independent, connection from the L. IFG to the L. IPS, as well as that from the L. IPS to the R. IPS, would provide a feedforward signal, together with negative feedback connections. We indicate that mathematics and language share the network of the L. IFG and L. IPS/SMG for the computation of hierarchical tree structures, and that mathematics recruits the additional network of the L. IPS and R. IPS.

## Introduction

One of the fundamental properties common to language and mathematics is the critical involvement of tree structures in those comprehension and production processes. Indeed, sentences consist of hierarchical tree structures with recursive branches [1], [2], and mathematical calculations can be also expressed by hierarchical tree structures [3], which may derive from the unique and universal property of recursive computation in humans [4]. This point provides a good motivation for the theoretical and experimental approaches advocated in the present study. According to modern linguistics, the construction of any grammatical phrase or sentence is based on the fundamental linguistic operation of “Merge,” which combines two syntactic objects to form a larger structure [5]. To properly measure the complexity of tree structures with a formal property of Merge and iterativity (recursiveness) [6], we recently introduced “the Degree of Merger (DoM),” which was defined as the maximum depth of merged subtrees (i.e., Mergers) within an entire sentence [7]. Among various models that may possibly quantify the complexity of tree structures, “number of nodes” would be a straight-forward model, simply counting the total number of non-terminal nodes (branching points) and terminal nodes of a tree structure. However, this model cannot capture hierarchical levels within the tree (sister relations in linguistic terms), whereas the DoM plays a critical role in measuring hierarchical levels of tree structures, such that the same numbers are assigned to the nodes with an identical hierarchical level [8]. Therefore, the model of DoM can properly capture recursiveness in the whole tree structures. Here we apply the computational concept of DoM to tree structures in mathematical calculations, and we hypothesize that the DoM actually represents specific loads in the computation of hierarchical tree structures also in mathematics.

Generally speaking, mathematical calculations consist of at least two components. One component is “mathematical syntax” that determines how terms and operators are combined together to form mathematical expressions. We hypothesize that mathematical syntax is shared with linguistic syntax in a deeper sense, and that both syntax can be automatically processed without consciousness or verbalization. The other component is “mathematical semantics” that deals with the meaning of terms, operators, and mathematical expressions. There have been a number of lesion studies and imaging studies, which claimed that linguistic and arithmetic abilities were separable in the brain [9]–[16]. However, they examined participants'abilities in elementary processing of numbers or variables, i.e., mathematical *semantics*, but not those in mathematical syntax. On the other hand, common activation between linguistic and arithmetic tasks may not necessarily mean that the same neural system subserves the same processes included in both tasks, because even a single region may have multiple functions. A superficial comparison of activated regions between linguistic and arithmetic tasks cannot resolve this critical issue. Here we propose a direct test, based on our previous finding that activations in the left inferior frontal gyrus (L. IFG) and the left supramarginal gyrus (L. SMG) were parametrically modulated by the DoM in a linguistic task; indeed, the DoM turned out to be the best factor among the 19 models tested [7]. If activations in the same regions are exactly modulated by the DoM alone in an arithmetic task, then the results would indicate functional commonality between linguistic and arithmetic computation. We thus focus on mathematical syntax, and our goal is to clarify whether the computation of hierarchical tree structures in mathematics and language share the same neural network.

Controversial issues still remain concerning the involvement of the L. IFG in mathematics. It has been proposed in a lesion study that the L. IFG is a shared substrate between sentence comprehension and arithmetic calculations [17], while arithmetic and algebraic calculations seemed to be preserved in some agrammatic patients [18], [19]. However, the knowledge about brackets and operators for determining the order of calculations does not guarantee effective computation of hierarchical tree structures in mathematical expressions. Moreover, when the patients were allowed to explicitly write partial results of individual calculations for either given or generated brackets, as in the case of the latter studies, individual calculations could be performed without actual construction of hierarchical tree structures for the whole expression. Likewise, overlapping activation in the L. IFG has been reported between sentence comprehension and arithmetic calculations [20], while other researchers opposed the involvement of the L. IFG in mathematics [21]. Regarding these imaging experiments, various factors including memory-related factors and applications of arithmetic operations (here denoted as “number of operations”; e.g., +, ×, etc.) may have influenced cortical activation. Therefore, the effect of the DoM should be segregated from other factors involved.

**Figure 1:**
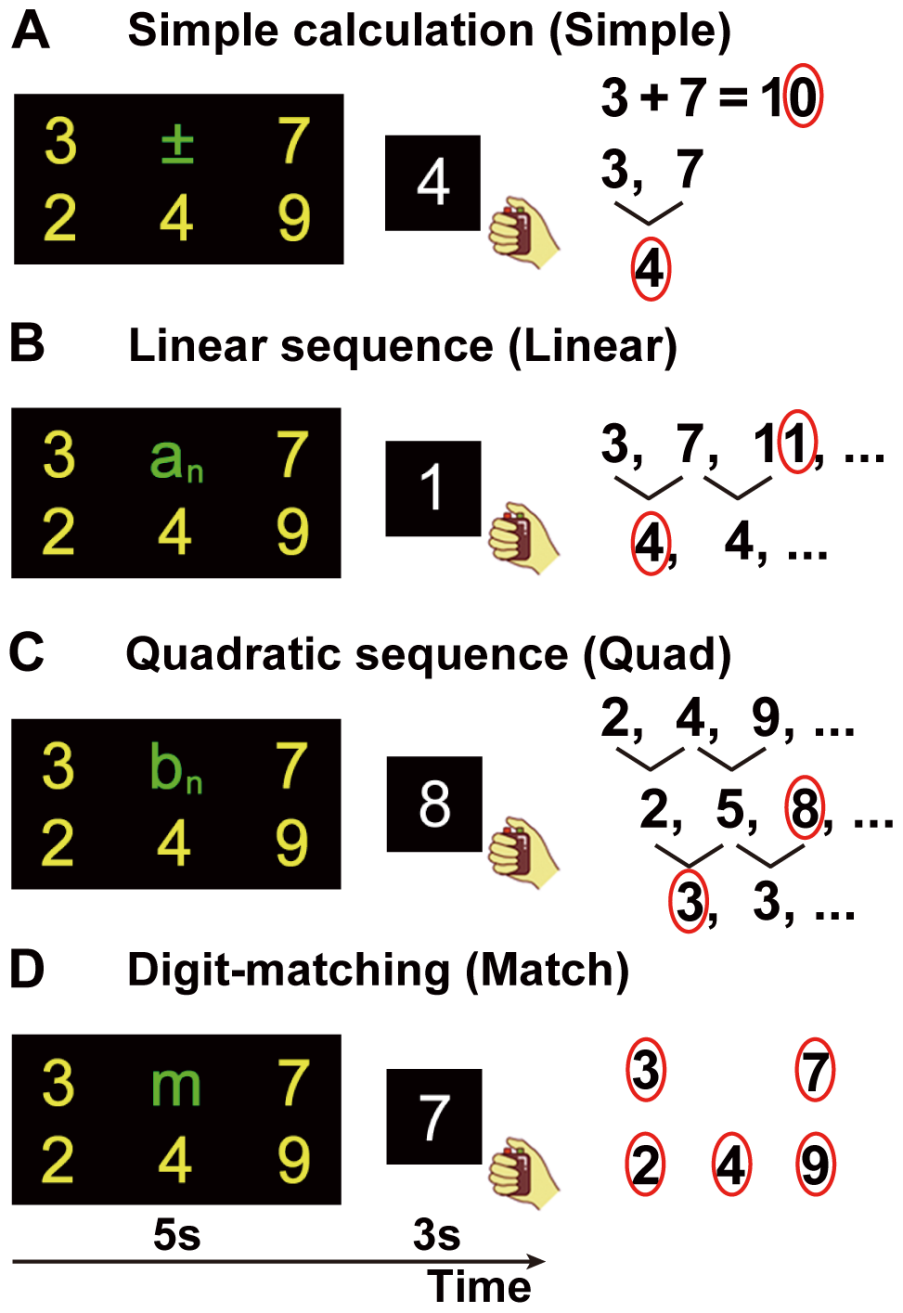
Examples of the stimuli used in the arithmetic task and Match task. Five yellow digits, together with a green character as a cue, were presented for 5 s, followed by the presentation of one white digit (matching stimulus) for 3 s. For each example, the task-relevant digits and a brief outline of the calculation processes are shown in the right panels, where the digits circled in red are the digits stored in memory for digit-matching. The participants judged whether one of those stored digits appeared as a white digit; all white digits shown here are correct examples. (A) Under the Simple condition, indicated by the presentation of “±” as a cue, the addition and subtraction of the upper two digits (i.e., two of the five yellow digits) were mentally performed by the participants. We instructed the participants to always subtract a smaller value from a larger value. (B) Under the Linear condition, indicated by the presentation of “a*_n_*” as a cue, the third term of a linear sequence initiated by the upper two digits was calculated by the participants. The first row at the top of the right panel represents the linear sequence, and the second row with branches represents the *constant* differences between each pair of adjacent terms in the linear sequence, as often used in math textbooks. (C) Under the Quad condition, indicated by the presentation of “b*_n_*” as a cue, the following arithmetic calculations were performed by using the lower three digits (i.e., three of the five yellow digits). The first row in the right panel represents the given quadratic sequence. The second row represents the differences between each pair of adjacent terms in the given sequence. The participants were asked to regard the resultant second row as a linear sequence, whose third term was then calculated in the same manner as the Linear condition. (D) In the Match task, indicated by the presentation of “m” as a cue, the participants stored all of the five digits in memory.

**Figure 2:**
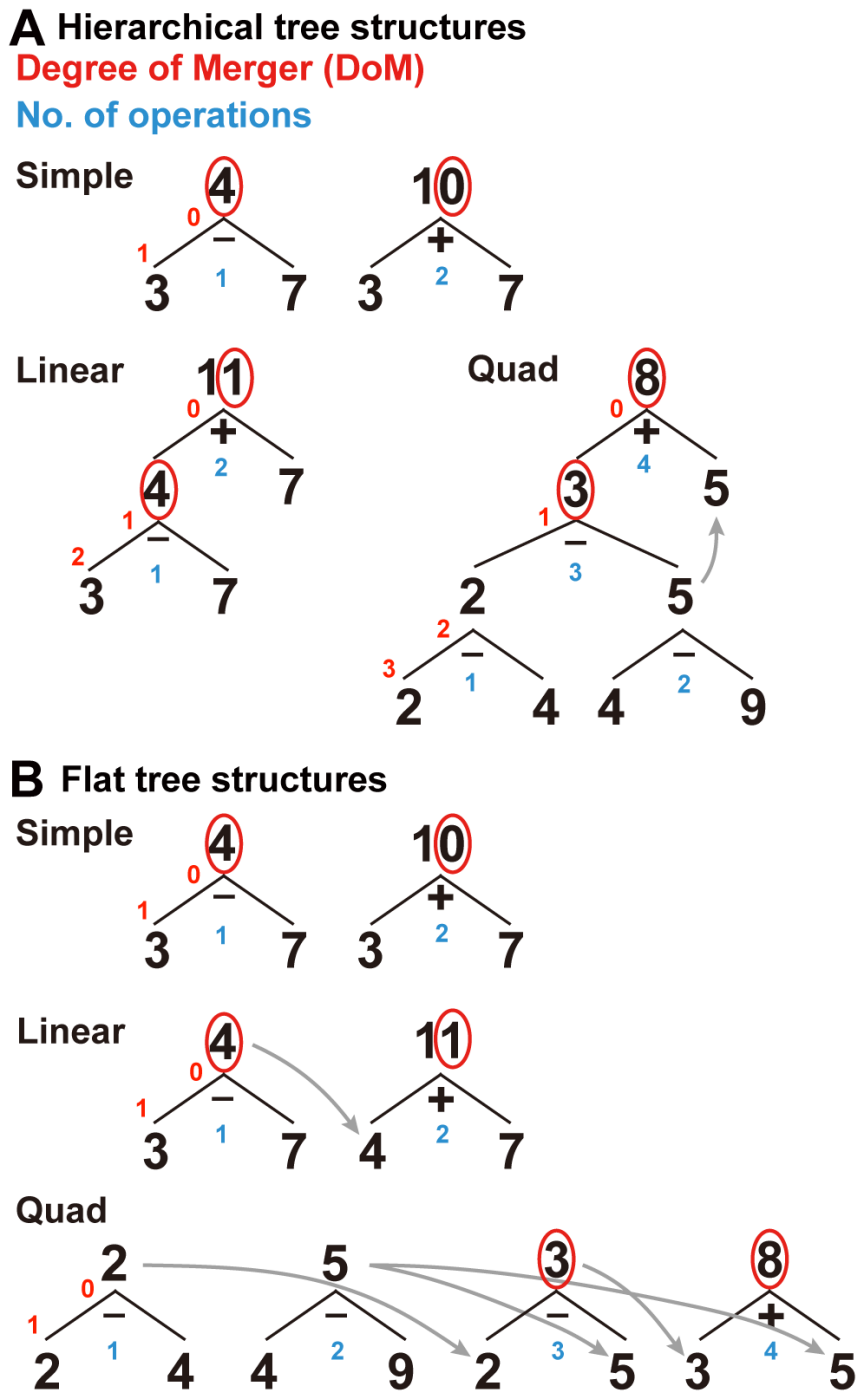
Hierarchical and flat tree structure models for arithmetic calculations. For each example shown in Figure 1, an entire calculation process is represented by either hierarchical (A) or flat (B) tree structure models, where the lowest and leftmost branch corresponds to the first arithmetic calculation performed. A digit in black at each nonterminal node is obtained from an arithmetic operation (e.g., + or −) indicated just below each node, where ‘–’ denotes a single operation of subtracting a smaller value from a larger value. The digits in red denote the DoM at individual nodes, where a reference point of zero is at the top node. The digits in blue denote “number of operations.” A gray arrow denotes corresponding digits generated temporarily in a calculation. The stored digits are circled in red, as shown in Figure 1. In the hierarchical tree structure model, each tree structure is based on recursive computation. Under the Linear condition, the hierarchical representation of the given example is: [|3−7| +7]  = 11. Under the Quad condition, the hierarchical representation of the given example is: [||2−4|− |4–9|| +5]  = 8.

In the present functional magnetic resonance imaging (fMRI) study, we prepared a novel arithmetic task, in which participants performed a series of specified arithmetic calculations without writing or overtly verbalizing partial results, and they stored two digits in memory for matching. We presented five digits, and tested three conditions in the arithmetic task: simple calculation (Simple, capitalized to indicate a condition name), linear sequence (Linear), and quadratic sequence (Quad) conditions. Under the Simple condition, the participants were asked to mentally perform the addition (e.g., 3+7 = 10), as well as the subtraction, of the upper two digits (Figure 1A). Under the Linear condition, the participants were asked to regard the upper two digits (e.g., 3 and 7) as a part of a linear sequence in increasing order (3, 7, 11, 15, 19,… in this case), in which the differences between each pair of adjacent terms were constant (Figure 1B). The participants were instructed to calculate the third term of the linear sequence. We employed linear sequences, because it naturally imposed recursive computation; e.g., 3, 7, 11, 15, 19,…, obtained by *nested* constructions ((((3+4) +4) +4) +4 …). The arithmetic task could not be correctly performed without generating such nested constructions to integrate new terms in a linear sequence. Under the Quad condition, the participants were asked to regard the lower three digits (e.g., 2, 4, and 9) as part of a quadratic sequence in increasing order (2, 4, 9, 17, 28,… in this case), in which the differences between each pair of adjacent terms resulted in a *subordinate linear sequence* (2, 5, 8, 11,… in this case) (Figure 1C). The participants were instructed to calculate the third term of the subordinate linear sequence. We theoretically predicted that this generative process imposed effective computation and actual construction of hierarchical tree structures (Figure 2A), just like the generative process of integrating new words in a sentence. As a basic control, we used a digit-matching (Match) task, in which the participants simply stored five digits in memory (Figure 1D).

Under each of the three conditions in the arithmetic task, we examined which of a hierarchical tree structure model or a flat tree structure model could properly explain the results. A linear sequence tested under the Linear condition internally combined arithmetic calculations of addition and subtraction tested under the Simple condition, whereas a quadratic sequence (e.g., 1, 2, 5, 10, 17,…) tested under the Quad condition internally involved a subordinate linear sequence (e.g., 1, 3, 5, 7,…). In addition, the Match task had no arithmetic calculation. Based on the hierarchical tree structure model (Figure 2A), the idea behind such nested task designs was to linearly increase the DoM in the hierarchical tree structures, i.e., “1, 2, and 3” under the Simple, Linear, and Quad conditions, respectively (see the row of the DoM under “Factors in the hierarchical tree structures” in Table 1). On the other hand, it is possible that the participants covertly verbalized each individual calculation process with digits and operations (e.g., “7−3 = 4”). If such individual calculations were represented by the flat tree structure model (Figure 2B), the DoMs became all “1” under the three conditions (see the row of the DoM under “Factors in the flat tree structures” in Table 1). However, the relationships among the generated digits became unnecessarily complex in the flat tree structure model (compare gray arrows under the Quad condition in Figure 2A and 2B). We predict that recursive computation in both linguistic and arithmetic processes automatically employs hierarchical tree structures; this argument closely resembles an argument over hierarchical versus linear-order representation of a sentence, e.g., “[[*To be happy*] *is fun*]” similar to [|3−7|+7].

**Table 1:** Estimates of various factors to account for activations.

Factors in the hierarchical tree structures	Factor	Simple	Linear	Quad	Match
	DoM	1	2	3	0
	No. of nodes	6	5	9	5
		**Simple** ***–*** **Match**	**Linear ** ***–*** **Match**	**Quad ** ***–*** **Match**	**Linear ** ***–*** **Simple**
	DoM	1	2	3	1
	No. of nodes	1	**0**	4	**−1**

Factors in the flat tree structures	Factor	Simple	Linear	Quad	Match
	DoM	1	1	1	0
	No. of nodes	6	6	12	5
	Verbal encoding	8	8	16	5
		**Simple** ***–*** **Match**	**Linear ** ***–*** **Match**	**Quad ** ***–*** **Match**	**Linear ** ***–*** **Simple**
	DoM	1	1	1	**0**
	No. of nodes	1	1	7	**0**
	Verbal encoding	3	3	11	**0**

Common factors	Factor	Simple	Linear	Quad	Match
	No. of operations	2	2	4	0
	No. of generated digits for calculation	0	1	4	0
	No. of stored digits for matching	2	2	2	5
		**Simple** ***–*** **Match**	**Linear ** ***–*** **Match**	**Quad ** ***–*** **Match**	**Linear ** ***–*** **Simple**
	No. of operations	2	2	4	**0**
	No. of generated digits for calculation	**0**	1	4	1
	No. of stored digits for matching	**−3**	**−3**	**−3**	**0**

We defined the estimate of a factor as the largest value that the factor can take for each condition: e.g., “1, 2, 3, and 0” for the DoM in the hierarchical tree structures. For each factor, its unit load should be invariable among all conditions, making an independent subtraction between estimates of the same factor possible. We assumed that positive and negative values of the subtracted estimates corresponded to activations and deactivations, respectively. Under all tested conditions, “number of operations” was equal to “number of Merge,” which was the total number of binary branches in the tree structures (Figure 2). Null or negative subtracted estimates were denoted in bold.

We fitted parametric models of eight factors (Table 1) to activations in each identified region, and determined the most crucial factor accounting for activations. The operational definitions of these factors other than the DoM are as follows. “Number of nodes” was equal to the total number of digits that appeared in each tree structure. For the stimuli used in the Match task, there were five terminal nodes without nonterminal nodes. The factor of “verbal encoding” in the flat tree structures was the total number of all possible digits and operations verbalized for individual calculations, which corresponded to the addition of two factors: “number of nodes” in the flat tree structures and “number of operations.” We also considered three common factors that were applicable to both types of tree structures. First, “number of operations” was equated between the Simple and Linear conditions. Secondly, “number of generated digits for calculation” was the number of digits generated temporarily in a calculation (i.e., not available on screen). For an example under the Quad condition shown in Figure 2B, there were four generated digits used in this calculation, “2, 5, 3, and 5.” Thirdly, “number of stored digits for matching” was the number of memorized digits that were used for digit-matching. Its estimate was two and five for the arithmetic task and the Match task, respectively (the digits circled in red in Figure 1 and 2).

By taking the Match task as a basic control for the arithmetic task, we eliminated any task-related cognitive factors, such as visual processing of stimuli, the identification of presented digits, digit-matching, and motor responses. Both “number of generated digits for calculation” and “number of stored digits for matching” might contribute to loads of short-term memory or “working memory,” but the Simple – Match contrast was free from these memory-related factors, because their subtracted estimates were null or negative (see Table 1). Moreover, the Linear – Simple contrast was also free from the following factors: “number of nodes” in either tree structures, the DoM and “verbal encoding” in the flat tree structures, “number of operations,” and “number of stored digits for matching.” The Quad – Linear contrast provides no further information, since no additional factors can be controlled. To examine the functional specialization of cortical regions in an unbiased manner [22], we adopted whole-brain analyses in such stringent contrasts as Simple – Match and Linear – Simple.

Recent neuroimaging studies have examined functional connectivity underlying elementary calculations. For example, it has been reported that the magnitude of functional connectivity between the bilateral intraparietal sulcus (IPS) correlated with the performances of a subtraction task [23]. Another fMRI study with Granger causality mapping reported that a participant group with high arithmetic scores in a multiplication task had stronger bidirectional connections between the L. IPS and R. IPS than the group with low arithmetic scores [24]. Both of those connectivity studies with elementary calculation tasks did not involve a recursive application of arithmetic operations. The effective connectivity during arithmetic tasks with recursive computation, as well as the connectivity between the L. IFG and bilateral IPS, should be thus clarified. In the present study, we adopted dynamic causal modeling instead of Granger causality mapping, because dynamic causal modeling has been shown to be more effective for fMRI data [25], [26]. Our findings would elucidate the crucial network of the L. IFG, L. IPS, and R. IPS for computing hierarchical tree structures in mathematics.

## Materials and Methods

### Participants

Twenty college students (15 males, aged 18–30 years), who had not majored in mathematics but studied high school mathematics including linear and quadratic sequences, participated in the fMRI experiment. All participants were healthy and right-handed (laterality quotient: 50–100), according to the Edinburgh inventory [27]. Prior to their participation in the study, written informed consent was obtained from each participant after the nature and possible consequences of the studies were explained. Approval for the experiments was obtained from the institutional review board of the University of Tokyo, Komaba.

### Stimuli

In each trial (Figure 1), we presented a main stimulus consisting of five yellow digits (from 1 to 9) and a green character (±, a*_n_*, b*_n_*, or m). Each green character was used as a cue to indicate one of the four conditions: “±” for the Simple condition, “a*_n_*” representing a linear sequence for the Linear condition (e.g., a*_n_* = 1, 3, 5, 7,…), “b*_n_*” representing a quadratic sequence for the Quad condition (e.g., b*_n_* = 1, 2, 5, 10,…), and “m” for the Match task. According to a pilot study, the duration of 5 s for the main stimulus was long enough for the participants to correctly perform the task. At the center of the screen, one white digit (from 0 to 9) was subsequently presented for 3 s as a matching stimulus, followed by a 200 ms blank to make the duration of the trial twice as long as the repetition time of the fMRI scans.

To make the stimuli physically identical among the three conditions in the arithmetic task, except for the cue characters, we used the same set of main stimuli, in which the five digits were always arranged in two rows (48 different combinations of digits). The upper two digits were relevant to the Simple and Linear conditions, while the lower three digits were relevant to the Quad condition. The digits in each row were arranged in increasing order from left to right, while no digit appeared twice in an entire stimulus. For the upper two digits, we excluded such ubiquitous combinations as “2 and 4,” “3 and 6,” or “4 and 8,” as well as trivial combinations with the constant difference of one (e.g., “2 and 3”). For the lower three digits, we excluded certain combinations (e.g., “1, 2, and 4”), in which the subordinate linear sequence became trivial (1, 2, 3, 4,… in this case). In the Match task, all digits in both rows were memorized; we prepared 72 different combinations of digits, including all combinations used in the arithmetic task.

The participants wore earplugs and an eyeglass-like MRI-compatible display (resolution, 800×600 pixels; VisuaStim Digital, Resonance Technology Inc., Northridge, CA). For fixation, a small red cross was always shown at the center of the screen to initiate eye movements from the same fixed position, and the participants were instructed to return their eyes to this position for the matching stimulus. Reaction times were measured from the onset of the matching stimulus. The stimulus presentation and collection of behavioral data (accuracy and reaction times) were controlled using the LabVIEW software and interface (National Instruments, Austin, TX).

### Tasks

In each trial of the arithmetic task, the participants were asked to silently perform a series of specified arithmetic calculations, and to store two digits in memory obtained from the arithmetic calculations without using their fingers. The participants then judged whether or not one of the stored digits matched the white digit, and responded by pressing one of two nonmagnetic buttons (Current Designs, Inc., Philadelphia, PA): a right button if matched (in half of the trials), or a left button if mismatched (in the other half).

Under the Simple condition (Figure 1A), the participants stored the results of addition and subtraction in memory. When the result was a “two-figure number” (e.g., 10), the participants simply stored the *last* digit (i.e., the digit in “the one's place” in math terms, 0 in this case) in memory. We instructed them to always subtract a smaller value from a larger value (e.g., |3−7|  = 4), as represented by the sign of absolute value here. Under the Linear condition (Figure 1B), the participants were instructed to obtain the constant difference first by the subtraction (e.g., |3−7|  = 4), and then to calculate the *third term* by adding the constant difference to the second term (4+7 = 11 in this case); they stored the results of these arithmetic calculations (4 and 1 in this case) in memory. Under the Quad condition (Figure 1C), the participants were instructed to perform a series of subtractions (e.g., |2−4|  = 2, and then |4−9|  = 5) in order to obtain the constant difference of the subordinate linear sequence by the subtraction (|2−5|  = 3 in this case). They were further instructed to calculate the *third term* of the subordinate linear sequence by adding the constant difference to the second term (3+5 = 8). They stored the results of these arithmetic calculations (3 and 8 in this case) in memory.

In the Match task (Figure 1D), the participants simply stored all of five yellow digits in memory, and judged whether one of those digits matched the white digit. The participants underwent practice sessions for these arithmetic and Match tasks before scanning. Eight scanning sessions were performed in one day. A single scanning session contained 36 trials (six trials for each of the Simple, Linear, and Quad conditions; 18 trials for the Match task). The arithmetic and Match tasks were alternately performed, so that MR signals for each condition in the arithmetic task were sufficiently separated. In the arithmetic task, no stimuli appeared twice or more times across all scanning sessions, whereas each stimulus appeared twice in the Match task. To prevent any condition-specific strategy, the order of the Simple, Linear, and Quad conditions was pseudorandomized; the orders of tasks and conditions were also counter-balanced across participants.

### MRI Data Acquisition

The fMRI scans were conducted on a 3.0 T scanner (Signa HDxt; GE Healthcare, Milwaukee, WI) with a bird-cage head coil. For the fMRI, we scanned 40 axial slices that were 3-mm thick with a 0.3-mm gap, covering from −52.8 to 78.9 mm from the anterior to posterior commissure line in the vertical direction, using a gradient-echo echo-planar imaging sequence [repetition time  = 4.1 s, echo time  = 60 ms, flip angle  = 90°, field of view  = 192×192 mm^2^, resolution  = 3×3 mm^2^]. In a single scanning session, we obtained 72 volumes following four dummy images, which allowed for the rise of the MR signals. For each participant, sessions without head movement were used for analyses; the number of abandoned sessions was less than four for all participants. After completion of the fMRI sessions, high-resolution T1-weighted images of the whole brain (192 axial slices, 1×1×1 mm^3^) were acquired from all participants with a fast spoiled gradient recalled acquisition in the steady state sequence (repetition time  = 8.4 ms, echo time  = 2.6 ms, flip angle  = 25°, field of view  = 256×256 mm^2^).

### fMRI Data Analyses

We performed data analyses with fMRI using SPM8 statistical parametric mapping software (Wellcome Trust Centre for Neuroimaging, London, UK; http://www.fil.ion.ucl.ac.uk/spm/) [28], implemented on a MATLAB platform (MathWorks, Natick, MA). The acquisition timing of each slice was corrected using the middle slice as a reference for the echo-planar imaging data. We realigned the echo-planar imaging data in multiple sessions to the first volume in all sessions, and removed sessions that included data with a translation of>2 mm in any of the three directions and with a rotation of>1.4° around any of the three axes; these thresholds were empirically determined from our previous studies [29]–[31].

Each participant's T1-weighted structural image was coregistered to the mean functional image generated during realignment. The coregistered structural image was spatially normalized to the standard brain space as defined by the Montreal Neurological Institute using the “unified segmentation” algorithm with medium regularization, which is a generative model that combines tissue segmentation, bias correction, and spatial normalization in the inversion of a single unified model [32]. After spatial normalization, the resultant deformation field was applied to the realigned functional imaging data, which was resampled every 3 mm using seventh-degree B-spline interpolation. All normalized functional images were then smoothed by using an isotropic Gaussian kernel of 9 mm full-width at half maximum. Low-frequency noise was removed by high-pass filtering at 1/128 Hz.

In a first-level analysis (i.e., a fixed-effects analysis), each participant's hemodynamic responses induced by the trials were modeled with a box-car function (band-pass during 1–3 s after the main stimulus onset) convolved with a hemodynamic function. The 0–1 s period from the main stimulus onset was related with the identification of a cue, and the 3–5 s period was likely to be confounded with preparatory processes of digit-matching. We thus selected the 1–3 s period in order to focus on arithmetic calculations. Only event-related responses of correct trials were analyzed. The images of the three conditions in the arithmetic task and Match tasks were then generated in a general linear model for each participant, and used for intersubject comparisons in a second-level analysis (i.e., random-effects analysis). To discount any general effects associated with task difficulty or performance differences among the participants, individual error rates (100 – accuracy), which were more sensitive than reaction times in our tasks, were entered for each task as a nuisance factor. For all fMRI data analyses, the statistical threshold was set to *P*<0.05 for the voxel level, corrected for multiple comparisons [family-wise error correction] across the whole brain.

For the anatomical identification of activated regions, we basically used the Anatomical Automatic Labeling method [33]. In region of interest analyses, we extracted the percent signal changes averaged among participants at each local maximum using the MarsBaR-toolbox (http://marsbar.sourceforge.net/). To statistically evaluate the fitness of a single factor's parametric model to activations, we calculated the coefficient of determination (*r* ^2^) and a residual sum of squares with MATLAB; we obtained the fitted values by multiplying the estimates of the factor (see Table 1) by a fitting scale. For a no-intercept model, *r* ^2^ = 1− Σ(*y*−*ŷ*)^2^/Σ*y* ^2^ should be calculated, where *ŷ* and *y* denote the fitted values and the *observed* signal changes for each contrast, respectively [34]. For this calculation, we used R software (http://www.r-project.org/). To further take account of individual variability, we used a restricted maximum-likelihood method, fitting “linear mixed-effects models” with individual activations as dependent variables, with the estimates of each factor as a regressor, and with the participants as random effects. For this calculation, we used an nlme (linear and nonlinear mixed-effects models) package (http://cran.r-project.org/web/packages/nlme/) on R software.

### Dynamic Causal Modeling Data Analyses

We performed data analyses with dynamic causal modeling using DCM10 on SPM8 [35]. We concatenated the scans from the separate sessions, and reanalyzed the preprocessed data with the general linear model, which contained two regressors representing the Quad condition and Match task for making a meaningful contrast, as well as a regressor representing both the Quad and Linear conditions for driving inputs. The regressor representing the Quad condition was also used for a modulatory effect. The effects of transition between sessions were taken into account with regressors of sessions. With the volume-of-interest tool in SPM8, the time series was extracted by taking the first eigenvariate across all suprathreshold voxels in Quad – Match for each participant (uncorrected *P*<0.05), confined within 6 mm from the group local maximum of a single region.

We specified 18 models with systematic variations in a modulatory effect and driving inputs (Figure S1). After estimating all models for each participant, we identified the most likely model by using random-effects Bayesian model selection on DCM10. Inferences from Bayesian model selection can be based on the expected probability, i.e., the expected likelihood of obtaining the model for any randomly selected participants, or on the exceedance probability, i.e., the probability that the model is a better fit to the data than any other models tested. After determining the best model, we evaluated the parameter estimates of this particular model by one-sample *t*-tests [36].

## Results

### Behavioral Results

The behavioral data are shown in Table 2, indicating that the tasks were performed almost perfectly. With respect to accuracy, a one-way repeated measures analysis of variance showed a significant main effect of condition including the Match task [*F*(3, 57)  = 20, *P*<0.0001]. Post-hoc paired *t*-tests among all conditions (significance level at *α* = 0.0083, Bonferroni-corrected) showed that the accuracy under the Quad condition was significantly lower than that under the other conditions (*P*<0.001), while the differences of accuracy among the other three conditions were not significant (*P*>0.1). Regarding reaction times (RTs), a main effect of condition was significant [*F*(3, 57)  = 12, *P*<0.0001], and the RTs under the Quad condition was significantly longer than those under the Simple condition (*P*<0.001). The Match task was also more demanding than the Simple and Linear conditions, leading to significantly longer RTs (*P*<0.001).

**Table 2:** Behavioral data under each condition.

	Simple	Linear	Quad	Match
Accuracy (%)	97.6±2.7	98.5±2.9	92.6±5.0	98.8±1.4
RTs (ms)	838±151	850±150	895±174	931±172

Behavioral data (mean ± standard deviation) of the accuracy and reaction times are shown for each condition. Only correct trials were included for reaction times, which were measured from the onset of the matching stimulus.

### Selective Activation in the Arithmetic Task

**Figure 3:**
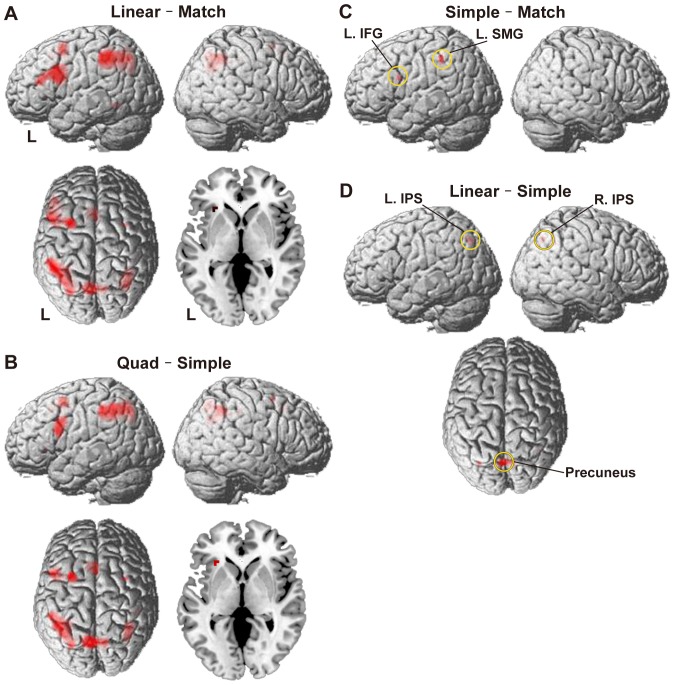
Significantly activated regions in the arithmetic task. Cortical activation maps are shown for the contrasts of Linear – Match (A), Quad – Simple, masked with Linear – Match (B), Simple – Match (C), and Linear – Simple (D). Activation is projected onto the left (L) and right lateral surfaces of a standard brain (family-wise error corrected *P*<0.05), as well as onto the dorsal surface where there was significant activation. A transverse plane at *z* = 1 shows activation in the left anterior short insular gyrus. See Tables 3 for the stereotactic coordinates.

To identify cortical regions involved in the arithmetic task, we first tested the Linear – Match contrast with the liberal control of Match. We then tested the direct Quad – Simple contrast, inclusively masked with Linear – Match to guarantee the consistency of activation patterns. In both contrasts, overall activation was clearly left-dominant. Significant activation was observed in the L. IFG (Brodmann's areas 44/45), bilateral SMG (Brodmann's area 40), bilateral IPS (Brodmann's areas 7/39/40), and precuneus (Brodmann's area 7), as well as in the bilateral lateral premotor cortex (LPMC, Brodmann's areas 6/8), left anterior short insular gyrus (ASG), and pre-supplementary motor area (pre-SMA, Brodmann's areas 6/8) (Figure 3A, 3B, and Table 3). The left middle temporal gyrus (L. MTG, Brodmann's area 21) was significantly activated only in Linear – Match.

**Table 3:** Significantly activated regions in the arithmetic task.

Contrast	Brain region	BA	Side	*x*	*y*	*z*	*Z*-Value	Voxels
Linear – Match	LPMC	6/8	L	*−*27	5	55	5.5	82
			R	33	5	58	4.5	5
	IFG	44/45	L	*−*45	11	25	6.8	309
				*−*51	32	22	5.2	*
	ASG	*–*	L	*−*30	26	1	4.6	5
	pre-SMA	6/8	M	*−*6	20	46	6.0	53
	SMG	40	L	−45	*−*40	43	6.9	483
	IPS	7/39/40	L	−33	*−*52	40	6.8	*
	SMG	40	R	48	*−*31	46	4.5	5
	IPS	7/39/40	R	36	*−*55	43	6.0	169
	Precuneus	7	M	*−*6	*−*70	46	6.1	155
	MTG	21	L	*−*51	*−*55	*−*11	5.2	15
Quad – Simple, masked with Linear – Match	LPMC	6/8	L	*−*24	5	55	5.4	62
			R	30	5	58	5.6	5
	IFG	44/45	L	*−*45	5	37	5.9	131
				*−*45	11	25	5.4	*
	ASG	*–*	L	*−*30	23	1	6.1	5
	pre-SMA	6/8	M	*−*6	20	43	6.4	51
				0	11	55	6.1	*
	SMG	40	L	−48	*−*37	43	5.4	352
	IPS	7/39/40	L	−33	*−*49	40	5.8	*
				−27	*−*70	43	5.5	*
	SMG	40	R	45	*−*31	43	5.9	5
	IPS	7/39/40	R	39	*−*52	43	5.4	110
				33	*−*61	34	4.8	*
	Precuneus	7	M	*−*12	*−*61	52	6.3	155
				9	*−*70	49	6.2	*
Simple – Match	IFG	44/45	L	−45	11	25	4.6	5
	SMG	40	L	*−*48	*−*37	46	4.8	12
Linear – Simple	IPS	7/39/40	L	−30	*−*70	46	4.6	3
			R	39	*−*55	43	4.5	4
	Precuneus	7	M	*−*3	*−*70	43	4.7	28

Stereotactic coordinates (*x, y, z*) in the Montreal Neurological Institute space (mm) are shown for each activation peak of *Z*-values (corrected *P*<0.05). LPMC, lateral premotor cortex; IFG, inferior frontal gyrus; ASG, anterior short insular gyrus; pre-SMA, pre-supplementary motor area; SMG, supramarginal gyrus; IPS, intraparietal sulcus; MTG, middle temporal gyrus; BA, Brodmann's area; L, left hemisphere; R, right hemisphere; M, medial. The region with an asterisk is included within the same cluster shown one row above.

We tried to narrow down the critical regions for the computation of hierarchical tree structures, by using more stringent contrasts. In Simple – Match, where the memory-related factors of “number of generated digits for calculation” and “number of stored digits for matching” were eliminated, significant activation was localized in the L. IFG and L. SMG (Figure 3C and Table 3), indicating that activations in these regions were free from the memory-related factors. Moreover, in Linear – Simple, significant activation was observed in the bilateral IPS and precuneus (Figure 3D and Table 3), indicating that activations in these regions were independent from other possible factors. Although activations in the L. IFG and L. SMG were below the threshold in Linear – Simple, they were significant with small volume correction (corrected *P*<0.05, 9 mm radius from each local maximum determined by Simple – Match). In Simple – Match with small volume correction, activations in the L. IPS, but not those in the R. IPS or precuneus, were significant (9 mm radius from each local maximum determined by Linear – Simple). These results suggest that the L. IFG, L. SMG, and L. IPS, together with the limited contribution of the R. IPS and precuneus, are well specialized in arithmetic calculations.

### Cortical Activations Specifically Modulated by the DoM in the Hierarchical Tree Structures

**Figure 4:**
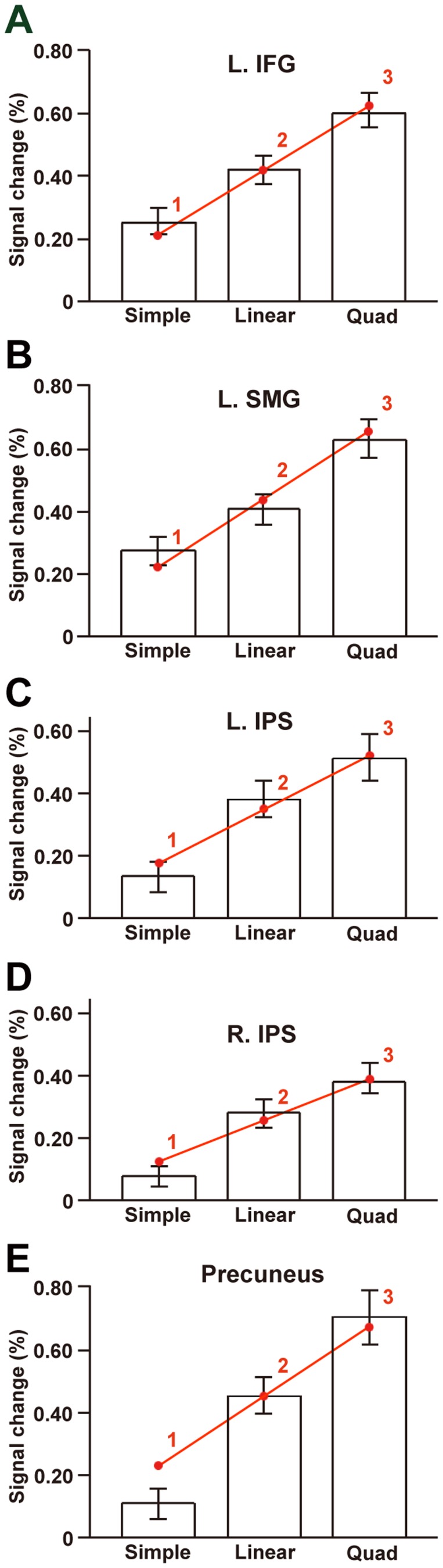
Quantified activations modulated by the DoM in the hierarchical tree structures. The percent signal changes in the L. IFG (A) and L. SMG (B) were taken from the local maxima in Simple – Match (circled in yellow in Figure 3C), whereas those in the L. IPS (C), R. IPS (D), and precuneus (E) were taken from the local maxima in Linear – Simple (circled in yellow in Figure 3D). Activations are shown for the Simple, Linear, and Quad conditions with reference to the Match task. Error bars indicate the standard error of the mean for the participants (*N* = 20). Overlaid red dots and lines denote the values fitted with the estimates (digits in red) for the DoM in the hierarchical tree structures (see Table 4).

At the local maxima of these five regions, we further examined percent signal changes under each condition in the arithmetic task with reference to the Match task (Figure 4). A linear modulation of activations was observed in the L. IFG, L. SMG, and bilateral IPS among these three conditions, indicating that the results were consistent with the DoM in the hierarchical tree structures. The precuneus showed weaker activations under the Simple condition, exhibiting relatively larger responses under the more demanding Linear and Quad conditions (Figure 4E).

Next we examined how well activations in each of the L. IFG, L. SMG, bilateral IPS, and precuneus correlated with the DoM in the hierarchical tree structures and other factors. All of the Simple – Match, Linear – Match, and Quad – Match contrasts predicted that activations should be exactly zero when a factor produced no effect or load relative to the Match task. We thus adopted a no-intercept model, in which the percent signal changes of each region were fitted with a single (thus minimal) scale parameter to a model of each factor using its subtracted estimates (Table 4). For the three contrasts, a least-squares method was used to minimize the residual sum of squares for the three fitted values (i.e., the three estimates multiplied by the fitting scale) against corresponding signal changes averaged across participants (Table 5, Tables S1-S4 in File S1).

**Table 4:** The results of fitted scale and values for each activated region, by using “DoM in the hierarchical tree structures”.

Brain region	Fitted scale	Fitted values
L. IFG	0.21	0.21, 0.41, 0.62
L. SMG	0.21	0.21, 0.42, 0.63
L. IPS	0.17	0.17, 0.34, 0.51
R. IPS	0.13	0.13, 0.26, 0.38
Precuneus	0.22	0.22, 0.45, 0.67

We obtained fitted values by multiplying the estimates of “1, 2, and 3” (see Table 1) by the fitting scale. The three fitted values correspond to activations observed under the Simple, Linear, and Quad conditions (see Figure 4).

**Table 5:** Fittings and likelihood of various models tested in the L. IFG.

Factors in the hierarchical tree structures	Factor	RSS	*r* ^2^	*P*-values	Log-likelihood	Likelihood ratio
	*DoM	0.0024	> 0.99	0.34, 0.75, 0.97	15.0	1
	No. of nodes	0.18	0.70	< 0.0001, 0.054, 0.65	*–*20.5	3.9 × 10*^–^* ^16^

Factors in the flat tree structures	Factor	RSS	*r* ^2^	*P*-values	Log-likelihood	Likelihood ratio
	DoM	0.062	0.90	0.0016, 0.0025, 0.85	*–*2.5	2.6 × 10*^–^* ^8^
	No. of nodes	0.13	0.78	< 0.0001, 0.0033, 0.20	*–*15.8	4.4× 10*^–^* ^14^
	Verbal encoding	0.063	0.89	< 0.0001, 0.14, 0.17	*–*6.7	3.7 × 10*^–^* ^10^

Common factors	Factor	RSS	*r* ^2^	*P*-values	Log-likelihood	Likelihood ratio
	No. of operations	0.015	0.98	0.033, 0.21, 0.68	9.6	0.0044
	No. of generated digits for calculation	0.13	0.78	< 0.0001, < 0.0001, 0.24	*–*15.0	9.1 × 10*^–^* ^14^
	No. of stored digits for matching	0.60	0	< 0.0001, < 0.0001, < 0.0001	n/a	n/a

Percent signal changes in the L. IFG were fitted with a single scale parameter to a model of each factor using its subtracted estimates (Table 1) for Simple – Match, Linear – Match, and Quad – Match. The *P*-values for the *t*-tests between the fitted value for each contrast and individual activations are shown in ascending order. Note that the model of DoM in the hierarchical tree structures (with an asterisk) resulted in the best fit for this region, i.e., with the least residual sum of squares (RSS), largest coefficient of determination (*r* ^2^), and larger *P*-values. The likelihood of models with all null estimates was not calculable (n/a). A likelihood ratio is the ratio of each model's likelihood to the best model's likelihood. The best model of DoM in the hierarchical tree structures was by far more likely than the other models.

Among the parametric models of eight factors tested, the model of DoM in the hierarchical tree structures produced by far the lowest residual sum of squares value (≤0.0043) and the largest coefficient of determination (*r* ^2^) (≥0.99) for the L. IFG, L. SMG, and bilateral IPS. We further evaluated the goodness of fit for each model by using one-sample *t*-tests (significance level at *α* = 0.0167, Bonferroni-corrected) between the fitted value for each contrast and individual activations. The model of DoM for these four regions produced no significant deviation for the three contrasts (*P*≥0.13). In the precuneus, the goodness of fit was marginal for one *P*-value (*P* = 0.027) under the Simple condition. By fitting “linear mixed-effects models,” we found that the model of DoM was by far most likely to explain the modulation of activations for all five regions (Table 5, Tables S1–S4 in File S1). Evidently, all of the other factors were clearly less effective than the DoM in the hierarchical tree structures.

### Effective Connectivity among the L. IFG and Bilateral IPS

**Figure 5:**
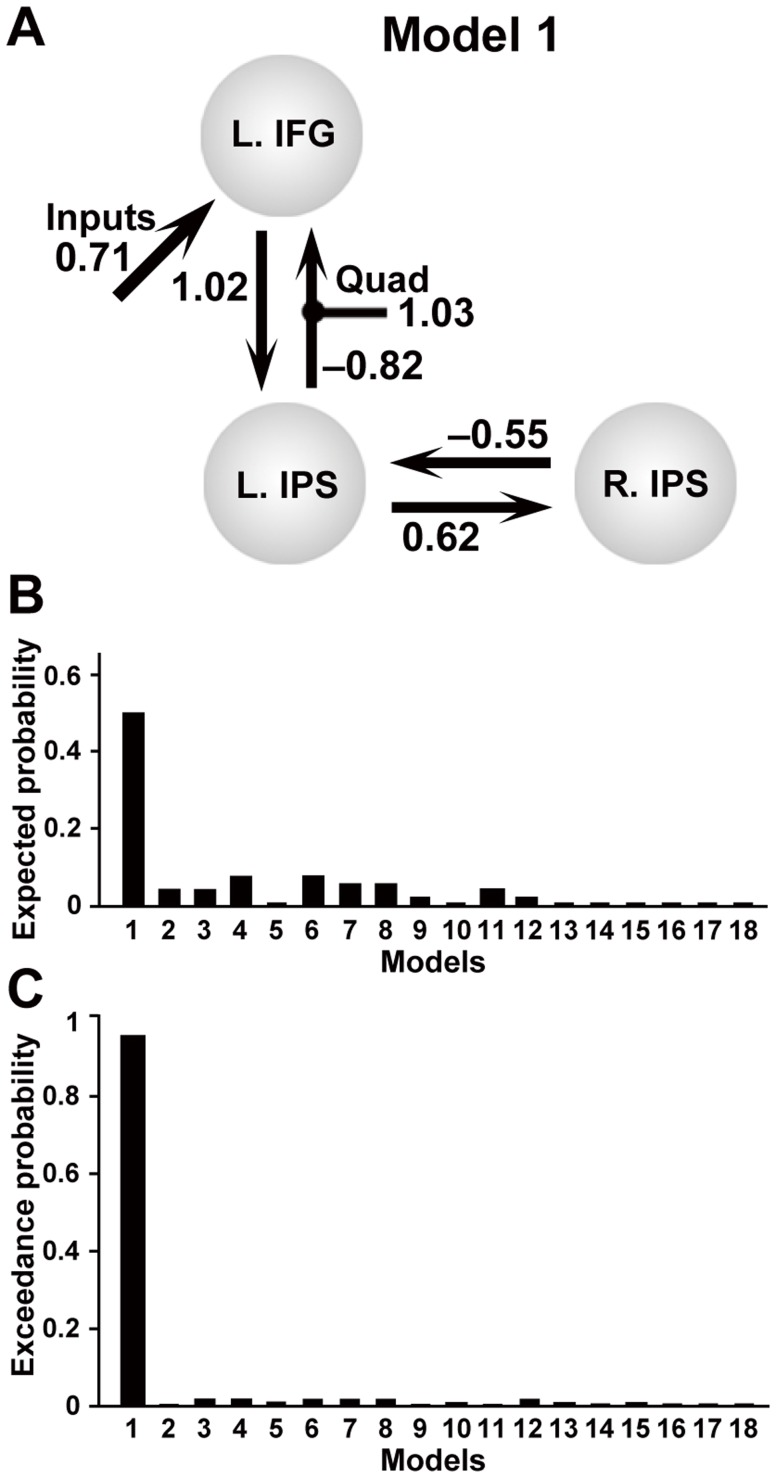
Effective connectivity among the L. IFG and bilateral IPS. (A) The best model with a positive modulatory effect for the bottom-up connection from the L. IPS to the L. IFG, and with driving inputs into the L. IFG. Mean parameter estimates that exceeded the statistical threshold (corrected *P*<0.05) are indicated alongside the intrinsic connections. Bar graphs show expected probabilities (B) and exceedance probabilities (C) of all models tested (Figure S1).

Because the L. SMG is relatively close to the L. IPS, and the precuneus locates midway between the bilateral IPS, we focused on the L. IFG and bilateral IPS alone for modeling the effective connectivity in the dynamic causal modeling analyses. Here we assumed intrinsic, i.e., task-independent, bidirectional connections between the L. IFG and L. IPS, as well as between the bilateral IPS. We systematically constructed 18 models with driving inputs into one of these three regions, such that for each input type we tested six models with a modulatory effect under the Quad condition on the unidirectional or bidirectional connections (see Figure S1 for all models tested). Using a random-effects Bayesian model selection, we showed that the model 1 (Figure 5A), with a modulatory effect for the connection from the L. IPS to the L. IFG, and with driving inputs into the L. IFG, yielded by far the highest expected probability and exceedance probability (Figure 5B and 5C).

For this best model, we further tested whether the parameter estimates were significantly different from zero among the participants. The intrinsic connection from the L. IFG to the L. IPS [1.02, *t*(19) = 8.9, *P*<0.001], and that from the L. IPS to the R. IPS were significantly positive [0.62, *t*(19) = 7.0, *P*<0.001]. In contrast, the intrinsic connection from the L. IPS to the L. IFG [−0.82, *t*(19) = 5.4, *P*<0.0001], and that from the R. IPS to the L. IPS were significantly negative [*−*0.55, *t*(19) = 3.4, *P*<0.005] (significance level at *α* = 0.0125; Bonferroni-corrected within a parameter class of intrinsic connections). The positive modulatory effect for the connection from the L. IPS to the L. IFG [1.03, *t*(19) = 6.2, *P*<0.001], as well as the driving inputs into the L. IFG [0.71, *t*(19) = 6.0, *P*<0.001], were also significant.

## Discussion

Here we introduced the DoM to the hierarchical tree structures in mathematics, and we obtained three striking results. First, we found significant activation in the L. IFG, L. SMG, bilateral IPS, and precuneus selectively among the three conditions in the arithmetic task (Figure 3). Secondly, by examining percent signal changes in each region, a linear modulation of activations was observed in the L. IFG, L. SMG, and bilateral IPS among these three conditions (Figure 4). Moreover, by fitting the parametric models of eight factors, we found that the DoM in the hierarchical tree structures best explained the modulation of activations in the L. IFG, L. SMG, bilateral IPS, and precuneus (Table 5, Tables S1–S4 in File S1). These results indicate the existence of mathematical syntax processed in these regions, excluding the load on “working memory.” The dominance of the hierarchical tree structure model is consistent with our previous results of the L. IFG and L. SMG activation in the direct comparison between sentences and letter strings, which were assumed to have hierarchical tree structures and flat tree structures, respectively [7]. Thirdly, using dynamic causal modeling, we showed that the model with a modulatory effect for the connection from the L. IPS to the L. IFG, and with driving inputs into the L. IFG, was highly probable (Figure 5). For this best model, the top-down intrinsic connection from the L. IFG to the L. IPS, as well as that from the L. IPS to the R. IPS, would provide a feedforward signal with their reverse connections representing a negative feedback signal. These results indicate that the network of the L. IFG and bilateral IPS subserves the computation of hierarchical tree structures in mathematics.

Previous imaging studies have established that syntactic processes during sentence comprehension selectively activate the L. IFG and/or the L. LPMC [29], [37]–[41]. By directly contrasting a demanding condition for “working memory,” we have previously demonstrated that both regions are indeed independent from such domain-general cognitive factors [29], indicating that these regions have a critical role as a putative grammar center [42]. It has been a matter of controversy whether the L. IFG is also critically involved in mathematics. It was claimed in a previous study that “it [the L. IFG] does not appear to play a dominant role [in mathematics], which instead is taken up by fusiform, parietal and precentral cortices,” i.e., visuo-spatial areas [21]. In this previous study, the participants performed a short-term matching task, which was solved without requiring any arithmetic calculation. In contrast, Makuuchi et al. [20] showed overlapped activation across sentence comprehension and arithmetic calculations, with an increase of activations in the L. IFG for the higher level of structural hierarchy, although the modulation of activations in the bilateral IPS were not fully examined. The present results clearly showed that the DoM in the hierarchical tree structures was by far more effective than the load on “working memory,” “number of operations,” and other factors for explaining the L. IFG and bilateral IPS activations.

According to some previous imaging studies, the domain-specificity for the arithmetic calculation in the L. IFG and other regions has remained unclear. For example, in recent fMRI studies [43], [44], two conditions with large and small numbers were compared in an addition task, and such domain-general factors as task difficulty might explain the enhanced activations. It should be noted that any hierarchical processes associated with “two-figure numbers,” as well as more verbal encoding, are also involved in the task with large numbers. In the present study, we discounted any general effects associated with task difficulty by entering individual error rates for each task as a nuisance factor. We have previously demonstrated that the L. IFG is a domain-specific neural system for syntactic computation in language, which is separable from other domain-general cognitive systems [42]. In the present study, we indicate that the hierarchical tree structures in mathematics are also computed by the same domain-specific system. Our successful approach on mathematical syntax can be naturally extended to “musical syntax” as well, since harmonic progressions are expressed by hierarchical tree structures [45].

Lesion studies have previously reported that the damage to the L. IPS or R. IPS caused deficits in elementary processes of numbers [46], [47]. For example, a lesion in the R. IPS was associated with deficits in performing even simple subtraction with one-figure numbers [47]. Moreover, activation in the bilateral IPS has been frequently observed in fMRI studies on numerosities, digits, and elementary calculations [48]–[50]. Unconscious repetition priming of numbers (e.g., from “NINE” to “9”) caused the activation suppression in the bilateral IPS [51]. The repetition suppression and recovery for the deviant number (e.g., “50” versus repeated numbers around “18”) in these regions were also independent from notation changes (e.g., dots to digits or digits to dots) [52]. A recent fMRI study has reported that high school arithmetic scores correlated negatively with activations in the R. IPS during an elementary calculation task, while a positive correlation was observed in the L. SMG [53]. The L. IPS and adjacent L. SMG are also involved in language, especially in vocabulary knowledge or lexical processing [54], [55], as well as in searching syntactic features [7]. Taken these and present results together, the L. IPS/SMG would be also involved in both mathematics and language. As regards the precuneus, its activation has been reported in the previous fMRI studies on number comparisons or arithmetic calculations [56], [57]. Our results suggest that the R. IPS and precuneus support the L. IPS/SMG under such demanding conditions as the Linear and Quad conditions.

Using a visual picture-sentence matching task, we have recently tested twenty-one patients with a left frontal glioma, and found abnormal overactivity and/or underactivity in 14 syntax-related regions [58]. By examining the functional and anatomical connectivity among those regions, we have clarified three syntax-related networks. The network I (syntax and its supportive system) consists of the opercular/triangular parts of the L. IFG, L. IPS, right lateral frontal regions, pre-SMA, and right temporal regions, which were overactivated in the patients with a glioma in the L. LPMC. The network II (syntax and input/output interface) consists of the L. LPMC, left angular gyrus, lingual gyrus, and cerebellar nuclei, which were overactivated in the patients with a glioma in the opercular/triangular parts of the L. IFG. The network III (syntax and semantics) consists of the left ventral frontal and posterior temporal regions, which were underactivated in the patients with a glioma in the opercular/triangular parts of the L. IFG. Among the activated regions in the present study (Table 3), the L. IFG, L. IPS, R. LPMC, R. IFG, and pre-SMA are included in the network I, whereas the L. LPMC and L. MTG are included in the network II and network III, respectively. The overall activated regions for the arithmetic calculations thus share their functional roles with the syntax-related regions in language.

Our previous fMRI study revealed that the functional connectivity between the L. IFG and L. SMG was selectively enhanced during sentence processing [59]. A recent dynamic causal modeling study with a cross-modal picture-sentence matching task has suggested that the L. IFG received driving inputs and transferred that information to the temporal regions [60]. Our recent dynamic causal modeling study has suggested a top-down intrinsic information flow of syntactic processing from the L. IFG to the L. SMG, with driving inputs into the L. IFG [7]. This model is consistent with our present results of dynamic causal modeling, which further indicate that L. IPS activations mirrored a top-down influence regarding the DoM in the hierarchical tree structures computed in the L. IFG. For the bottom-up connection from the L. IPS/SMG to the L. IFG, the modulatory effect under the conditions with the largest DoM was *negative* in this previous study, whereas the modulatory effect under the Quad condition (with the largest DoM) was *positive* in the present study. While lexical feedback was minimum for processing jabberwocky sentences in the previous study, a positive feedback about operations would be utilized for constructing hierarchical tree structures in the present paradigm.

The present results of dynamic causal modeling suggest that the syntactic information on hierarchical tree structures provided in the L. IFG would be further processed through the top-down intrinsic connection from the L. IPS to the R. IPS (Figure 5). The L. IPS and R. IPS may have different roles in processing arithmetic calculations, but their individual roles in mathematical syntax should be clarified in the future studies. In addition, it is possible that the L. MTG, significantly activated in Linear – Match, is involved in mathematical semantics, as this region would subserve semantics in language. We indicate that mathematics and language share the network of the L. IFG and L. IPS/SMG for the computation of hierarchical tree structures, and that mathematics recruits the additional network of the L. IPS and R. IPS, with an information flow from the former to the latter.

## Supporting Information

Figure S1 **Models tested in the dynamic causal modeling analyses.** We assumed intrinsic, i.e., task-independent, bidirectional connections between the L. IFG and L. IPS, as well as between the L. IPS and R. IPS. Eighteen models were systematically constructed with driving inputs into one of the three regions. For each input type, we tested six models for the modulatory effect under the Quad condition.
(TIF)

File S1 Table S1: Fittings and likelihood of various models tested in the L. SMG. Table S2: Fittings and likelihood of various models tested in the L. IPS. Table S3: Fittings and likelihood of various models tested in the R. IPS. Table S4: Fittings and likelihood of various models tested in the precuneus.
(DOC)
