# Supplementary figures and images for: Neural Mechanisms Underlying the Computation of Hierarchical Tree Structures in Mathematics
Source: PLoS One. 2014 Nov 7;9(11):e111439. doi: 10.1371/journal.pone.0111439 (PMC4224410; doi:10.1371/journal.pone.0111439)

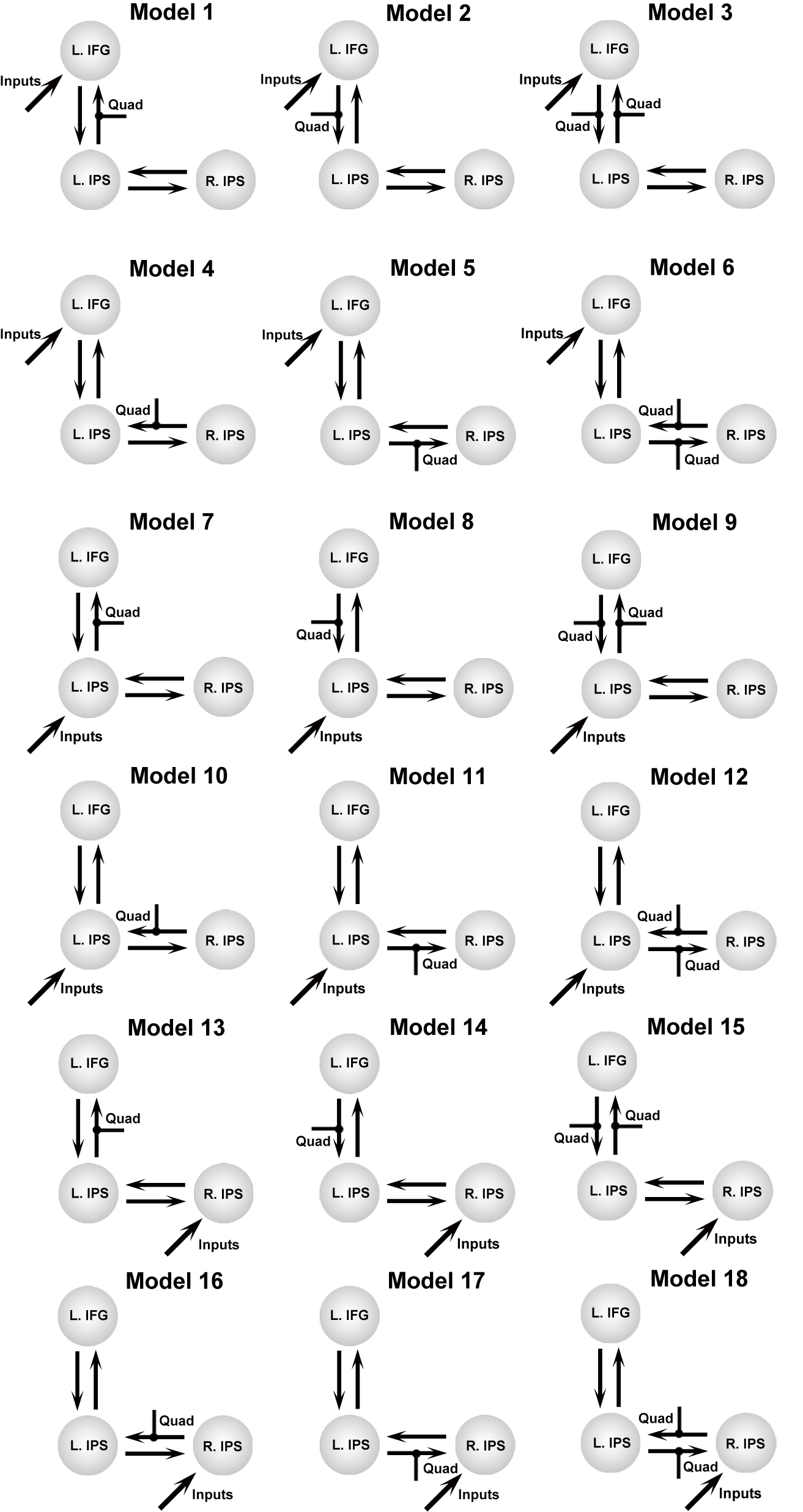

Supplement: Figure S1 — Models tested in the dynamic causal modeling analyses. We assumed intrinsic, i.e., task-independent, bidirectional connections between the L. IFG and L. IPS, as well as between the L. IPS and R. IPS. Eighteen models were systematically constructed with driving inputs into one of the three regions. For each input type, we tested six models for the modulatory effect under the Quad condition. (TIF) [file pone.0111439.s001.tif]
