# Supplementary material for: Neural Mechanisms Underlying the Computation of Hierarchical Tree Structures in Mathematics
Source: PLoS One. 2014 Nov 7;9(11):e111439. doi: 10.1371/journal.pone.0111439 (PMC4224410; doi:10.1371/journal.pone.0111439)
Supplement: File S1 — Table S1: Fittings and likelihood of various models tested in the L. SMG. Table S2: Fittings and likelihood of various models tested in the L. IPS. Table S3: Fittings and likelihood of various models tested in the R. IPS. Table S4: Fittings and likelihood of various models tested in the precuneus. (DOC) [file pone.0111439.s002.doc]

**Neural Mechanisms Underlying the Computation of Hierarchical Tree Structures in Mathematics**

**Tomoya Nakai, Kuniyoshi L. Sakai**

| **Table S1.** Fittings and likelihood of various models tested in the L. SMG. | | | | | | |
| --- | --- | --- | --- | --- | --- | --- |
|  | | | | | | |
| **Factors in the hierarchical tree structures** | **Factor** | **RSS** | ***r*^2^** | ***P*-values** | **Log-likelihood** | **Likelihood ratio** |
|  | *DoM | 0.0043 | 0.99 | 0.20, 0.69, 0.92 | 15.1 | 1 |
|  | No. of nodes | 0.17 | 0.72 | < 0.0001, 0.028, 0.68 | *–*23.3 | 2.2 × 10*^–^*^17^ |
| **Factors in the flat tree structures** | **Factor** | **RSS** | ***r*^2^** | ***P*-values** | **Log-likelihood** | **Likelihood ratio** |
|  | DoM | 0.064 | 0.90 | 0.0025, 0.0078, 0.54 | *–*6.0 | 6.9 × 10*^–^*^10^ |
|  | No. of nodes | 0.13 | 0.80 | < 0.0001, 0.0013, 0.31 | *–*19.3 | 1.2 × 10*^–^*^15^ |
|  | Verbal encoding | 0.056 | 0.91 | 0.0006, 0.097, 0.24 | *–*8.7 | 5.0 × 10*^–^*^11^ |
| **Common factors** | **Factor** | **RSS** | ***r*^2^** | ***P*-values** | **Log-likelihood** | **Likelihood ratio** |
|  | No. of operations | 0.0090 | 0.98 | 0.13, 0.31, 0.81 | 12.3 | 0.061 |
|  | No. of generated digits for calculation | 0.13 | 0.79 | < 0.0001, 0.0002, 0.39 | *–*19.3 | 1.2 × 10*^–^*^15^ |
|  | No. of stored digits for matching | 0.63 | 0 | < 0.0001, < 0.0001, < 0.0001 | n/a | n/a |
| In the L. SMG, the model of DoM in the hierarchical tree structures was by far more likely than the other models. | | | | | | |

| **Table S2.** Fittings and likelihood of various models tested in the L. IPS. | | | | | | |
| --- | --- | --- | --- | --- | --- | --- |
|  | | | | | | |
| **Factors in the hierarchical tree structures** | **Factor** | **RSS** | ***r*^2^** | ***P*-values** | **Log-likelihood** | **Likelihood ratio** |
|  | *DoM | 0.0032 | 0.99 | 0.36, 0.57, 0.92 | 7.1 | 1 |
|  | No. of nodes | 0.14 | 0.66 | < 0.0001, 0.98, > 0.99 | *–*23.4 | 6.1 × 10*^–^*^14^ |
| **Factors in the flat tree structures** | **Factor** | **RSS** | ***r*^2^** | ***P*-values** | **Log-likelihood** | **Likelihood ratio** |
|  | DoM | 0.075 | 0.82 | 0.0003, 0.027, 0.50 | *–*11.7 | 6.9 × 10*^–^*^9^ |
|  | No. of nodes | 0.092 | 0.78 | < 0.0001, 0.35, 0.50 | *–*18.8 | 5.7 × 10*^–^*^12^ |
|  | Verbal encoding | 0.053 | 0.87 | 0.0011, 0.46, 0.58 | *–*12.2 | 4.1 × 10*^–^*^9^ |
| **Common factors** | **Factor** | **RSS** | ***r*^2^** | ***P*-values** | **Log-likelihood** | **Likelihood ratio** |
|  | No. of operations | 0.031 | 0.92 | 0.0043, 0.082, 0.21 | *–*3.2 | 3.5 × 10*^–^*^5^ |
|  | No. of generated digits for calculation | 0.074 | 0.82 | 0.0007, 0.017, 0.42 | *–*16.2 | 8.0 × 10*^–^*^11^ |
|  | No. of stored digits for matching | 0.41 | 0 | < 0.0001, < 0.0001, 0.017 | n/a | n/a |
| In the L. IPS, the model of DoM in the hierarchical tree structures was by far more likely than the other models. | | | | | | |

| **Table S3.** Fittings and likelihood of various models tested in the R. IPS. | | | | | | |
| --- | --- | --- | --- | --- | --- | --- |
|  | | | | | | |
| **Factors in the hierarchical tree structures** | **Factor** | **RSS** | ***r*^2^** | ***P*-values** | **Log-likelihood** | **Likelihood ratio** |
|  | *DoM | 0.0029 | 0.99 | 0.14, 0.67, 0.93 | 25.1 | 1 |
|  | No. of nodes | 0.075 | 0.67 | < 0.0001, 0.58, 0.93 | *–*3.4 | 4.0 × 10*^–^*^13^ |
| **Factors in the flat tree structures** | **Factor** | **RSS** | ***r*^2^** | ***P*-values** | **Log-likelihood** | **Likelihood ratio** |
|  | DoM | 0.049 | 0.79 | < 0.0001, 0.098, 0.52 | 3.9 | 6.0 × 10*^–^*^10^ |
|  | No. of nodes | 0.047 | 0.80 | < 0.0001, 0.50, 0.61 | 1.5 | 5.5 × 10*^–^*^11^ |
|  | Verbal encoding | 0.028 | 0.88 | 0.0014, 0.26, 0.51 | 7.2 | 1.7 × 10*^–^*^8^ |
| **Common factors** | **Factor** | **RSS** | ***r*^2^** | ***P*-values** | **Log-likelihood** | **Likelihood ratio** |
|  | No. of operations | 0.020 | 0.91 | 0.0031, 0.057, 0.81 | 13.1 | 5.7 × 10*^–^*^6^ |
|  | No. of generated digits for calculation | 0.035 | 0.85 | 0.0010, 0.029, 0.41 | 5.1 | 2.1 × 10*^–^*^9^ |
|  | No. of stored digits for matching | 0.23 | 0 | < 0.0001, < 0.0001, 0.029 | n/a | n/a |
| In the R. IPS, the model of DoM in the hierarchical tree structures was by far more likely than the other models. | | | | | | |

| **Table S4.** Fittings and likelihood of various models tested in the precuneus. | | | | | | |
| --- | --- | --- | --- | --- | --- | --- |
|  | | | | | | |
| **Factors in the hierarchical tree structures** | **Factor** | **RSS** | ***r*^2^** | ***P*-values** | **Log-likelihood** | **Likelihood ratio** |
|  | *DoM | 0.014 | 0.98 | 0.027, 0.69, 0.93 | *–*5.90 | 1 |
|  | No. of nodes | 0.21 | 0.71 | < 0.0001, 0.20, 0.86 | *–*31.2 | 9.9 × 10*^–^*^12^ |
| **Factors in the flat tree structures** | **Factor** | **RSS** | ***r*^2^** | ***P*-values** | **Log-likelihood** | **Likelihood ratio** |
|  | DoM | 0.18 | 0.75 | < 0.0001, 0.0038, 0.60 | *–*28.1 | 2.2 × 10*^–^*^10^ |
|  | No. of nodes | 0.12 | 0.83 | < 0.0001, 0.57, 0.96 | *–*24.5 | 8.3 × 10*^–^*^9^ |
|  | Verbal encoding | 0.072 | 0.90 | 0.0003, 0.063, 0.63 | *–*18.5 | 3.4 × 10*^–^*^6^ |
| **Common factors** | **Factor** | **RSS** | ***r*^2^** | ***P*-values** | **Log-likelihood** | **Likelihood ratio** |
|  | No. of operations | 0.066 | 0.91 | 0.0002, 0.040, 0.58 | *–*16.1 | 3.8 × 10*^–^*^5^ |
|  | No. of generated digits for calculation | 0.084 | 0.88 | 0.0002, 0.031, 0.46 | *–*20.1 | 1.9 × 10*^–^*^9^ |
|  | No. of stored digits for matching | 0.72 | 0 | < 0.0001, < 0.0001, 0.031 | n/a | n/a |
| In the precuneus, the model of DoM in the hierarchical tree structures was by far more likely than the other models. | | | | | | |
